# The Relationship Between Nursing Practice Environment and Innovative Behavior in the Al-Madinah Region, Saudi Arabia: A Descriptive Study

**DOI:** 10.7759/cureus.31603

**Published:** 2022-11-17

**Authors:** Maryam A Alrefaei, Ghada M Hamouda, Ohood O Felemban

**Affiliations:** 1 Public Health Department/Nursing Administration, College of Nursing, King Abdulaziz University, Jeddah, SAU; 2 Public Health Department/Community and Primary Healthcare, College of Nursing, King Abdulaziz University, Jeddah, SAU

**Keywords:** saudi arabia, pes-nwi, nursing practice environment, nurses, innovative behavior, general hospitals, cross-sectional studies

## Abstract

Background and objective

A positive and supportive practice environment is essential for inspiring innovation in nursing. Innovative behaviors (IBs) could motivate nurses to devise solutions in several domains, such as identifying and solving workplace problems, building new work methods, delivering their services efficiently and effectively, adopting new medical technology advancements, and leading the change process to face current challenges in healthcare. In this study, we aimed to investigate the relationship between the nursing practice environment (NPE) and IB in the Al-Madinah region of Saudi Arabia.

Methods

A quantitative, descriptive, cross-sectional correlational design was employed for the study. The convenience sample consisted of 330 bedside nurses working in five general hospitals in the Al-Madinah region who voluntarily completed a self-report questionnaire consisting of queries related to demographic and professional characteristics, the Practice Environment Scale of the Nursing Work Index (PES-NWI), and the Innovative Behavior Inventory (IBI). The collected data were analyzed using descriptive statistics and Pearson’s correlation.

Results

Based on the study findings, the NPE was favorable. The overall PES-NWI mean score was 2.62 ± 0.50, and the mean scores of four of the five subscales were >2.50. The collegial nurse-physician relations subscale was perceived as the most favorable (2.87 ± 0.59), while staffing and resource adequacy was perceived as unfavorable (2.35 ± 0.65). The overall IBI mean score was 3.53 ± 0.56, indicating that nurses had a moderate level of agreement on IB. The highest mean score in IB was in the idea search domain (3.72 ± 0.77), while it was lowest in the implementation of starting activities domain (3.11 ± 0.86).

Conclusions

The correlation between the NPE and IB was positive and statistically significant. However, the correlation of the staffing and resources adequacy subscale in relation to subscales of idea search, overcoming obstacles, and innovation output did not reach statistical significance. Healthcare organizations should incorporate the principles of work innovation and healthy nursing work environments into their core values and enhance and nurture them through strategic management.

## Introduction

Healthcare delivery innovations could increase accessibility, efficacy, and affordability in today's changing healthcare systems characterized by advanced technology, limited resources, and empowered clients [1]. Saudi Arabia has embarked on a new era of development and innovation with the establishment of Saudi Vision 2030, a program that encourages and seeks advancements in different sectors that prioritize improving the healthcare delivery system to enhance community health [2]. Nurses should adapt to new ideas and advanced technology, foster progress, and provide patients with the best quality of care [3].

The terms nursing practice environment (NPE) and nursing work environment (NWE) are interchangeable when referring to organizational factors that support professional nursing practice [4]. The aspects of the NPE include autonomy, exemplary professional nursing practices, management support, personnel and resource sufficiency, and interdisciplinary team relationships [5]. The Practice Environment Scale of the Nursing Work Index (PES-NWI) provides a profile of the major domains in the NPE of the original Magnet hospital [4]. The PES-NWI scale was used to assess NPE in this study, as each domain can be amenable to interventions for improvement.

Another variable investigated in this study was innovative behavior (IB). Asurakkody and Shin [6] recently defined IB in the context of nursing as “generating novel ideas and putting effort to implement them with confidence, overcoming possible challenges to produce new procedures, treatment strategies, or policies for restoring and health promotion of patient or clients”. They also recognized eight dimensions of IB based on previous studies: (1) opportunity exploration; (2) idea generation; (3) idea search; (4) idea communication; (5) promotion of an idea; (6) idea championing; (7) application; and (8) overcoming obstacles. Previous research has found that the level of IB among nurses is moderate to high [3,7-9]. Notably, nurses with high levels of IB have a strong potential to succeed in their careers [3].

On the other hand, it should be mentioned that previous studies examining the IB of nursing employees were limited to examining the impact of some organizational characteristics [3,8,10-13]. However, the researchers have identified two recently published studies that examined the overall NPE in relation to IB [9,14]. Both studies have reported a statistically significant correlation between NPE and IB. Consequently, the authors have inferred that a favorable work environment could foster IB and vice versa. As long as the work environment's communications and processes are clear, with regular training and proper rewards and incentives, the IB of staff can be enhanced [9].

NPE must be constantly assessed in Saudi hospitals to guarantee its consistency with the Health Sector Transformation Program, which was launched to meet the requirements and achieve the objectives of Saudi Vision 2030. In addition, even though the IBs of nursing employees are recognized internationally as pivotal to the quality of healthcare services, to the best of our knowledge, no national studies have been conducted to analyze IBs within Saudi hospitals. Indeed, few studies have been conducted in the wider Middle East region to examine the relationship between NPE and IBs. Notably, no studies have been conducted on hospital nurses during the coronavirus disease 2019 (COVID-19) pandemic, making this research novel and unique. Of note, the COVID-19 pandemic has inspired healthcare professionals to innovate and overcome obstacles. Moreover, this study provides recommendations to hospital management, policymakers, and human resource managers to enhance organizational performance and productivity by fostering IBs.

## Materials and methods

Aim and design

This quantitative, descriptive, cross-sectional correlational study was conducted to investigate the relationship between NPE and IB in the Al-Madinah region of Saudi Arabia. This study aimed to address the following research questions:

1. What is the nurses' perception toward their practice environment characteristics?

2. What is the nurses' degree of agreement toward their IB?

3. Is there a relationship between the nurses' perception of NPE and IB in Al-Madinah government hospitals?

Sample and setting

Convenience sampling was used to recruit nurses from the five following government hospitals located in the Al-Madinah region: (1) King Fahad Hospital, with a 500-bed capacity; (2) Ohud Hospital, with a 253-bed capacity; (3) Al-Madinah Al-Munawarah Hospital, with a 200-bed capacity; (5) Yanbu General Hospital, with a 300-bed capacity; and (4) Khaybar General Hospital, with a 100-bed capacity. All selected hospitals were affiliated with the Saudi Ministry of Health (MOH) and provided multidisciplinary healthcare services. This study only included staff nurses with at least one year of experience in providing direct patient care in inpatient nursing units (critical and general departments) in the current work setting. Nurses in managerial positions, nurse educators, nurses who work in patient safety and total quality departments, and nurses who work in the outpatient or emergency departments were excluded from this study due to critical differences in terms of work conditions, job roles, and responsibilities. The sample size was calculated using Stephen Thompson’s formula based on the total number of nurses in the five hospitals (confidence interval = 95.0%, confidence limit = 0.05, N = 2,405).

$n=(N\ x\ p(1-p))/([[\ N-1x(d^2\div z^2\ )]+p(1-p)] )$

$n=\frac{2405x\ 0.50(1-0.50)}{2405-1x\left(\frac{0.0025}{3.84}\right)+0.50(1-0.50)}=331$

n: sample size (331); N: population size (2,405); Z: confidence level at 95% (1.96); d: error proportion (0.05); p: probability (50%)

Stratification was used to calculate the sample from each hospital as follows:

• Sample needed from Ohud Hospital = [546/2,405] x 331 = 75

• Sample needed from King Fahd Hospital = [881/2,405] x 331 = 121

• Sample needed from Al-Madinah Al-Munawwarah Hospital = [401/2,405] x 331 = 55

• Sample needed from Yanbu General Hospital = [467/2,405] x 331 = 64

• Sample needed from Khaybar General Hospital = [110/2,405] x 331 = 16

The sample size of the study was determined to be 331 registered nurses (RNs), recruited from selected hospitals as mentioned above. However, the final sample consisted of 330 bedside nurses, indicating a 99.7% response rate.

Data collection and instruments

The data collection process started on January 26, 2022, and was completed on March 29, 2022. Data were collected using a printed self-reported questionnaire administered in Arabic and English, which consisted of three main parts: (1) demographic and professional characteristics; (2) the PES-NWI [4]; and (3) IBI [15]. The questionnaire started with a cover letter that included the study title, study aim, target participants, estimated time to complete the questionnaire, and the researcher's contact information for any inquiry regarding the study.

Demographic and Professional Characteristics

The researchers developed this part of the questionnaire to assess the participants' basic demographic and professional characteristics. It incorporated seven items: gender, age, marital status, educational level, years of nursing experience, work setting, and working unit.

The Practice Environment Scale of the Nursing Work Index (PES-NWI)

The PES-NWI was used to assess nurses' perceptions of their current practice environment characteristics. The PES-NWI developed by Lake [4] was based on the characteristics of hospitals that were successful in attracting and retaining nurses during the nursing shortage crisis in the United States in the early 1980s. This scale comprises 31 items grouped under five subscales as follows: (1) nurse participation in hospital affairs (nine items); (2) nursing foundations for quality of care (10 items); (3) nurse manager ability, leadership, and support of nurses (five items); (4) staffing and resource adequacy (four items); and (5) collegial nurse-physician relations (three items). It was measured using a 4-point Likert scale ranging from 1 (strongly disagree) to 4 (strongly agree) to indicate whether the feature is present in the current work setting. The subscale score is the average of the subscale item responses. Values of 2.50 or greater imply a general agreement, whereas values less than 2.50 imply disagreement regarding the presence of the characteristics measured by the scales. NPE is classified into three categories based on the subscales' weighted mean: favorable, mixed, and unfavorable; practice environments are considered favorable if four or five subscales are above 2.50, mixed if two or three subscales are above 2.50, and unfavorable if zero to one subscale is above 2.50 [4].

Innovative Behavior Inventory (IBI)

IBI was used to assess nurses’ IBs. Lukes and Stephan [15] developed this scale in 2017, which comprises 23 items. The items are divided into seven domains as follows: (1) idea generation (three items); (2) idea search (three items); (3) idea communication (four items); (4) implementation starting activities (three items); (5) involving others (three items); (6) overcoming obstacles (four items); and (7) innovation outputs (three items). The IBI uses a 5-point Likert scale ranging from 1 (fully disagree) to 5 (fully agree). Higher values indicate a greater degree of IB among the respondents. The total IBI score was calculated as the mean score of the seven domains. In this study, IB was divided into three categories based on the total mean score. An IBI mean score of 4.00 or higher, equal to 3.00 and below 4.00, and less than 3.00 indicates that a nurse has a high, moderate, and low IB, respectively.

Validity and reliability of the tools

The PES-NWI has been shown to be a valid tool for measuring hospital NPEs [4]. Similarly, the IBI showed factorial, criterion, convergent, and discriminant validity, as well as cross-cultural equivalence [15]. In the current study, content validity was used to test the instrument's validity. The questionnaire was distributed to a panel of three experts specializing in nursing administration and public health. The expert panel recommended minor linguistic changes. Hence, the survey's translated version was modified prior to distribution to the participants. Cronbach's alpha coefficient for the original PES-NWI was reported as 0.82 for the entire scale, ranging from 0.71 to 0.84 for its subscales [4]. The scale is considered highly reliable and valid, with national and international studies reporting internal consistency reliability in the range of 0.83-0.94 [5,16-17]. In contrast, Cronbach's alpha values in the original IBI were found to vary between 0.60 and 0.88 [15], while it was found to be 0.92 in the study by Emiralioğlu and Sönmez [14] for the entire scale. In this study, Cronbach's alpha coefficient indicated the reliability of the PES-NWI (α = 0.887) and IBI (α = 0.904).

Data analysis

Data were coded and transferred by the researchers into a Microsoft Excel spreadsheet and then imported into SPSS Statistics version 21 (IBM Corp., Armonk, NY) for analyses. Descriptive statistics (frequency and percentage) were used to describe the sample demographics and professional characteristics, whereas the PES-NWI and IBI domains were presented as means and standard deviations. The Pearson correlation test was used to determine the correlation between the PES-NWI and IBI domains. A p-value of 0.05 was considered statistically significant, while a p-value of 0.01 was considered highly statistically significant.

Ethical considerations

All stages of this study were conducted in compliance with the fundamental ethical principles of the research. Prior to data collection, ethical approval was obtained from the Faculty of Nursing Research Ethics Committee at King Abdulaziz University (Ref no. 2M.81) and from the Institutional Review Board, General Directorate of Health Affairs in Madinah (IRB 152-2021). Permission to use the tools by the original authors was sought. The participant's right to privacy was protected by ensuring their anonymity. The cover letter stated that the questionnaire submission would be considered as providing consent for using their answers for research purposes (implied consent). In addition, the study had no risks to the participants due to the voluntary nature of participation, the right to withdraw from the study at any time, and the right to not complete the survey without inviting penalty or prejudicial treatment. Finally, the completed surveys were collected and secured by the researchers.

## Results

Demographic and professional characteristics

Of the 330 nurses who participated in this study, 47.6% were aged between 30-39 years, while 8.2% were over 40 years of age. In addition, 82.7% were females, and 58.8% were married. Regarding education, 62.1% had a bachelor’s degree in nursing, 34.8% had a diploma in nursing, and only 3.0% of the nurses held master’s degrees in nursing. Regarding years of nursing experience, 33% of nurses had less than five years of experience, while 7.0% had at least 15 years of experience. Nurses at King Fahad General Hospital represented 36.4% of the total sample. Lastly, 54.2% of the nurses worked in general departments, while 45.8% worked in critical care departments (Table 1).

**Table 1:** Distribution of nurses according to their demographic and professional characteristics (n = 330)

Demographic characteristics	Frequency (n = 330)	Percent (%)
Age group (years)		
20-29	146	44.2
30-39	157	47.6
≥40	27	8.2
Gender		
Male	57	17.3
Female	273	82.7
Marital status		
Single	117	34.8
Married	194	58.8
Divorced	19	5.8
Educational level		
Diploma	115	34.8
Bachelor's	205	62.1
Master's	10	3.0
Years of nursing experience		
<5	109	33.0
5-9	104	31.5
10-14	94	28.5
≥15	23	7.0
Work setting		
King Fahad General Hospital	120	36.4
Ohud Hospital	75	22.7
Al-Madinah General Hospital	55	16.7
Yanbu General Hospital	64	19.4
Khaybar General Hospital	16	4.8
Working unit		
Critical care department	151	45.8
General department	179	54.2

Nursing practice environment characteristics

A descriptive analysis (mean and standard deviation) of the data from the five subscales of PES-NWI was conducted to analyze nurses' perceptions of their practice environment characteristics. As shown in Table 2, out of the five subscales, nurses agreed on the presentation of four subscale characteristics in their practice environments. As illustrated, the two subscales that had the highest mean scores were “collegial nurse-physician relations” (2.87 ± 0.59) and “nursing foundations for quality of care” (2.75 ± 0.50). In contrast, “staffing and resource adequacy” (2.35 ± 0.65) and “nurse participation in hospital affairs” (2.51 ± 0.58) had the lowest mean scores. Lastly, based on this study's findings, the overall NPE was found to be favorable since the weighted mean of the four subscales was above 2.50.

**Table 2:** The mean score of nurses’ perceptions toward the five subscales of the nursing practice environment (n = 330) Mean scores: ≥2.50 indicates nurses’ agreement; <2.50 indicates nurses’ disagreement PES-NWI: The Practice Environment Scale of the Nursing Work Index; SD: standard deviation

PES-NWI domains	Mean ± SD
“Nurse participation in hospital affairs” total score	2.51 ± 0.58
“Nursing foundations for quality of care” total score	2.75 ± 0.50
“Nurse manager ability, leadership, and support of nurses” total score	2.64 ± 0.64
“Staffing and resource adequacy” total score	2.35 ± 0.65
“Collegial nurse-physician relations” total score	2.87 ± 0.59
Total PES-NWI score	2.62 ± 0.50

Nurses’ overall innovative behavior

Descriptive statistics of the IBI questionnaire and its domains are presented in Table 3. Based on the results, the highest mean score was in the “idea search” domain (3.72 ± 0.77), while the lowest was in the “implementation of starting activities” domain (3.11 ± 0.86). The overall mean score of the IBI questionnaire was 3.53 ± 0.56, which indicates that nurses had a moderate level of agreement on IB.

**Table 3:** The mean score of the IBI domains that reflected nurses’ innovative behavior (n = 330) Mean score: ≥4.00 indicates high agreement on innovative behavior; =3.00-<4.00 indicates moderate agreement on innovative behavior; <3.00 indicates low agreement on innovative behavior IBI: Innovative Behavior Inventory; SD: standard deviation

IBI domains	Mean ± SD
“Idea generation” total score	3.65 ± 0.78
“Idea search” total score	3.72 ± 0.77
“Idea communication” total score	3.61 ± 0.72
“Implementation of starting activities” total score	3.11 ± 0.86
“Involving others” total score	3.55 ± 0.78
“Overcoming obstacles” total score	3.51 ± 0.78
“Innovation output” total score	3.55 ± 0.69
Total IBI score	3.53 ± 0.56

Correlation between the nurses’ perception toward NPE and IB domains

As shown in Table 4, there was a significant positive correlation between the PES-NWI subscales according to the IBI domains for almost all variables, except for the correlation of “staffing and resource adequacy” in relation to “idea search” (r = 0.062), “overcoming obstacles” (r = 0.065), and “innovation output” (r = 0.099), and the correlation between “overcoming obstacles” and the IBI total score (r = 0.806), where the correlation did not reach statistical significance.

**Table 4:** Correlation (Pearson-r) between PES-NWI subscales and IBI domains (n = 330) I-V: The Practice Environment Scale of the Nursing Work Index (PES-NWI) subscales; VII-XIV: Innovative Behavior Inventory (IBI) domains **Correlation is significant at the 0.01 level (two-tailed). *Correlation is significant at the 0.05 level (two-tailed)

SN	Domains	I	II	III	IV	V	VI	VII	VIII	IX	X	XI	XII	XIII	XIV
I	Nurse participation	1													
II	Nursing foundation	0.770**	1												
III	Nurse manager ability	0.809**	0.680**	1											
IV	Staffing andf resource adequacy	0.682**	0.550**	0.681**	1										
V	Nurse-physician relations	0.561**	0.590**	0.476**	0.449**	1									
VI	PES-NWI total score	0.908**	0.846**	0.882**	0.822**	0.741**	1								
VII	Idea generation	0.214**	0.400**	0.214**	0.169**	0.210**	0.282**	1							
VIII	Idea search	0.158**	0.349**	0.129*	0.062	0.320**	0.238**	0.609**	1						
IX	Idea communication	0.351**	0.494**	0.312**	0.184**	0.388**	0.409**	0.509**	0.633**	1					
X	Implementation activities	0.189**	0.289**	0.236**	0.226**	0.240**	0.280**	0.306**	0.277**	0.510**	1				
XI	Involving others	0.281**	0.375**	0.338**	0.159**	0.298**	0.346**	0.407**	0.372**	0.592**	0.436**	1			
XII	Overcoming obstacles	0.198**	0.297**	0.190**	0.065	0.274**	0.247**	0.396**	0.572**	0.645**	0.440**	0.580**	1		
XIII	Innovation output	0.223**	0.258**	0.163**	0.099	0.231**	0.231**	0.328**	0.392**	0.456**	0.349**	0.420**	0.592**	1	
XIV	IBI total score	0.321**	0.484**	0.319**	0.194**	0.378**	0.403**	0.695**	0.744**	0.839**	0.656**	0.742**	0.809	0.669**	1

## Discussion

IB is pivotal to the quality of healthcare services. It is essential for staff nurses to be innovative in overcoming challenges that arise in healthcare settings and conduct evidence-based practice [11]. This study aimed to investigate the relationship between NPE and IB in the Al-Madinah region of Saudi Arabia. With regard to nurses' perceptions toward their practice environment characteristics, the study results showed that the NPE was favorable as perceived by nurses. The findings provide further validation of the results of a few national studies [17-18] and another international study in Brazil [19]. In contrast, other studies from Canada [20] and Korea [21] found that nurses rated their practice environments as mixed. On the other hand, a study conducted by Brofidi et al. [16] at five public hospitals in Greece showed that nurses assessed all five hospitals as unfavorable nursing practice environments. Interestingly, another study in Riyadh, Saudi Arabia [22], which included public and military hospitals and used the PES-NWI, found that nurses rated the NPE of the public hospitals as unfavorable. Only one subscale scored above 2.50, while the NPE of the military hospital was rated as favorable, and all five subscales scored above 2.50. The positive findings in the current study could be seen as a reflection of the MOH and the Saudi Government’s ongoing efforts to achieve “the gold standard” in nursing practice to meet the nursing goals outlined in the Saudi Vision 2030. In addition, nursing management emphasizing high nursing standards aligns with the health sector transformation program, which seeks to restructure the health sector in the country into a comprehensive, practical, and integrated health system based on the well-being of the individual and community.

Regarding the NPE characteristics in the study setting, the results showed that the “collegial nurse-physician relations” subscale had the highest agreement. This result is consistent with a study conducted in Canada by Smith et al. [23], where nurses gave the highest agreement score for the category of nurse-doctor relationship. Similarly, Brofidi et al. [16] mentioned that only this subscale was rated by nurses as positive among the five subscales representing NPE characteristics. The study results were expected, as the professions of nursing and medicine are called upon to work in exceptional proximity, not only side-by-side but also in collaboration, to accomplish the common aim of providing the best patient care. In addition, the Saudi MOH has created and enforced zero-tolerance policies to eliminate workplace abuse and other inappropriate behaviors.

Correspondingly, the current study’s findings showed that the lowest NPE subscale scores were marked for “staffing and resource adequacy”, followed by nurse participation in hospital affairs. The current findings revealed nurses' overall disagreement in terms of the presence of “staffing and resource adequacy” characteristics in their NPE. However, despite substantial efforts to make a difference, it is reasonable to connect the current results to the recently recorded obstacles in Saudi nursing practice. According to Alsufyani et al. [2], the current state of nursing practice in Saudi Arabia faces several obstacles, including a national nursing shortage that has led to heavy reliance on expatriate nurses. In addition, the rapid growth of the Saudi population and the constant changes in the healthcare system and health needs have aggravated the nursing shortage. In particular, public hospitals have a high patient-to-nurse ratio, as they cater to a large number of patients who seek free medical care. Moreover, the result could be attributed to the fact that public hospitals affiliated with the MOH have limited resources compared to other hospitals operated by the military or universities. Similarly, Brofidi et al. [16] confirmed that Greek nurses viewed the “staffing and resources adequacy” subscale as the most unfavorable organizational factor. In addition, Al Moosa et al. [18] reported that nurses perceived workforce adequacy as the least favorable subscale. However, the results of the present study were contradicted by the findings of a study conducted by Almuhsen et al. [24], who reported that the highest mean score among the five subscales was for staffing and resource adequacy. In addition, the results of the current study were incongruent with those of Phillips et al. [20], who found a high level of agreement for nurse staffing and resource-contributing variables among nurses in Alberta, Canada.

On the other hand, the current study found that nurses responded with a general agreement regarding the presence of the “nurse participation in hospital affairs” subscale in their NPE. This finding is consistent with that of previous studies by Al Moosa et al. [18], and Almuhsen et al. [24]. However, the average score was lower than that reported in previous studies. In contrast to these results, Brofidi et al. [16] reported that nursing professionals considered their participatory role unfavorable. The current findings could be attributed to the fact that despite the attempts of the MOH in Saudi Arabia to implement decentralized management models, the vast majority of healthcare decisions are still made by top-level management. Even unit-level nurse leaders have limited power, restricting their ability to affect the quality of NPEs [25]. Similarly, Alsufyani et al. [2] identified centralized management as an issue in Saudi Arabian nursing practice.

Furthermore, this study revealed that nurses generally agreed regarding the presence of “nursing foundations for quality of care” subscale characteristics in their organizations. This result is congruent with those of Al Moosa et al. [18], and Dordunoo et al. [21], who found that nurses reported highly positive perceptions toward this subscale. Ambani et al. [22] found that this subscale was the most favorable among the five NPE subscales. In contrast, a national study reported that nurses demonstrated overall disagreement with the presence of this subscale characteristic in their work environment [24]. This variation in findings may be attributable to the nature of the research population, which included nurse managers and staff nurses, or to institutional factors. Furthermore, the positive finding in this study could be explained by the fact that MOH hospitals provide a strong foundation for nurses to perform their responsibilities competently.

Likewise, the results of the present study confirmed nurses' overall agreement regarding the presence of characteristics outlined in the “nurse manager ability, leadership, and support of nurses” subscale in their organizations. This result is congruent with Al Moosa et al. [18] and Dordunoo et al. [21], who found that nurses reported highly favorable perceptions toward this subscale. Almuhsen et al. [24] mentioned that nurses disagreed with the factors reflecting this subscale in their practice environment. The current results could be explained by the fact that some head nurses in Saudi hospitals choose democratic leadership styles and maintain positive relationships with their subordinates, which may positively impact the quality of nursing care, contribute to conflict resolution, and reduce absenteeism. This explanation is endorsed by a study conducted by Dahshan et al. [26] in government hospitals in Taif in Saudi Arabia, which found that most leaders employed both transformational and transactional leadership styles.

As for the nurses' degree of agreement toward their IBs, the results of the current study revealed that nurses had a moderate level of agreement toward their IBs based on the average score of IBI domains. Due to a lack of national-level research evaluating IB among Saudi nurses, these results were compared to those from other countries and found to be consistent with studies in China [3], Egypt [8], and the United States [27]. In contrast, other studies from Turkey [14] and Egypt [9,28] reported high IB among nurses. The researchers hypothesized that the moderate level of agreement might be attributable to local hospital administrators' lack of awareness regarding the development of an innovative organizational culture. In such a culture, nurses are more likely to demonstrate IB because the organization encourages these behaviors by facilitating the production of new ideas and fostering a culture in which these ideas can be implemented. The “idea search” domain scored the highest among the seven IB domains. In contrast, the “implementation of starting activities” had the lowest mean scores. This result was in line with Mahgoub et al. [9], who demonstrated that the highest agreement among the domains of staff nurses' IB was in “innovation outputs” followed by “idea search” and “involving others”. Conversely, the “implementation of starting activities” domain had the least level of agreement. Interestingly, another study conducted at the Benha University Hospital in Egypt, which included nurses in three critical care units (intensive care unit, coronary care unit, and cardiothoracic care unit), found that idea championing and idea implementation had the highest mean scores among the IB domains [28].

Approximately half of the sample in this study are working in critical care areas, which could explain the highest rating for the “idea search” domain, as those nurses usually deal with patients requiring complex assessments and procedures, improving their capacity to think critically and invent new solutions. Additionally, they experience work-based problems on a daily basis, which prompts searches for existing knowledge sources in their environment and the creation of novel ideas and solutions for these problems. This explanation is supported by the American Association of Critical Care Nurses [29], which emphasizes that nurses who work in critical care units tend to be creative and lifelong learners who become more innovative over time and seek knowledge from different resources. Generally, nurses in all departments are closest to the point of care and are best positioned to seek optimal solutions. Dy Bunpin et al. [27] stated that RNs are in positions that require innovative clinical decision-making despite working in bureaucratic and hierarchical circumstances that may inhibit innovation.

On the other hand, the lowest rating in the “implementation of starting activities” domain could be explained by the fact that most nurses, particularly in general hospitals, may have remarkable innovative ideas but no strategies for implementation or do not take the necessary steps to make these ideas into reality due to inadequate resources, lack of administrative support, and the absence an organizational culture that values new ideas and encourages nurses to innovate in such hospitals. Dy Bunpin et al. [27] believed that RNs could play a leading role in assessing, establishing, implementing, adapting to, and evaluating new ideas, thereby contributing to improving patient outcomes. Asurakkody and Shin [6] argued that a key aspect of IB among nurses is to communicate ideas to management staff, whereas employees in any organization are rarely able to implement ideas on their own and must often obtain approval from managers.

Lastly, and most importantly, the results of this study revealed that there was a significant positive correlation between nurses' perceptions of their practice environment and IB. Indeed, the healthier and more supportive the NPE is, the more likely that nurses will behave in innovative ways. In other words, creating a favorable NPE characterized by the availability of information, resources, educational opportunities, effective teamwork, shared decision-making, management support, and work autonomy will motivate nurses to feel a need to reciprocate by engaging in IBs to perform their jobs effectively.

In addition, despite the dilemma of the constant shortage of staff and resources in the selected hospitals, staff nurses find it motivating to search for new solutions and valuable ideas to solve problems and obstacles in order to enhance the quality of healthcare provided. Therefore, this might be a possible explanation for this study's finding, as no correlations of the “staffing and resources adequacy” subscale with the “idea search”, “overcoming obstacles”, and “innovation output” domains were found. These findings are consistent with previous studies [9,14] in Turkey and Egypt, respectively, where nurses linked their IB to their NPE characteristics. Concurrently, other international studies have shown comparable results that reveal the association between the variables [7,11-12] and found that nurse managers’ leadership style, nursing staff's autonomy, adequate resources, and verbal encouragement had a positive impact on the IBs of nurses. Moreover, the results of this study are supported by Sönmez and Yıldırım [10], who found that inadequate time and resources negatively affect the development of IB, whereas supervisor support has a significant and positive effect on IB.

Study limitations

This study has a few limitations. The fact that the data were gathered through nurses' self-reports may have led the outcomes to be more positive, which, however, may have led to the risk of exaggeration or social desirability bias. Moreover, since this study involved a cross-sectional analysis of correlations, we cannot prove causality and must be careful not to assume that changes in the NPE caused changes in nurses’ IB. Another limitation is that the study was conducted exclusively in general hospitals with a convenience sample selected. This may have affected the results due to organizational culture and resource provision differences between these institutions. Lastly, the generalizability of the study's findings is limited because the healthcare systems and working environments in Saudi Arabian hospitals are diverse and depend on the ownership and operating system of each institution.

Recommendations related to nursing education

(i) It is suggested that nursing educators incorporate knowledge related to NPE to educate future nurses on how to recognize and foster favorable working conditions. Active RNs should be provided opportunities for ongoing training in creating safe and healthy workplaces.

(ii) Nursing educators need to run workshops on innovative thinking and design strategies to improve and facilitate IB among nurses. Also, they should develop courses related to increasing bedside nurses’ readiness to be change agents by staying abreast of the continual changes in healthcare systems.

Recommendations for further research

It could be worthwhile to explore the perspectives of nursing administrators on their IB and the individual and organizational aspects that impact it. Further studies utilizing mixed methods or qualitative approaches are recommended to obtain a broader knowledge of the correlation between NPE and IB among Saudi Arabian nurses. In addition, to strengthen the validity of the research findings and the generalizability of the results, it is necessary to replicate the study with a larger sample size across various institutions, such as private and military hospitals in Saudi Arabia. Finally, additional research should be undertaken using a longitudinal study design to ascertain the causal relationship between the main variables.

## Conclusions

Despite the limitations mentioned above, this study fills a gap in the existing literature by determining which characteristics of the NPE are mainly linked with IB among nurses in Saudi Arabia since no other study has examined the relationship between the two variables in Saudi hospitals. Therefore, nursing managers and top hospital management should consider bedside nurses' perceptions of the overall practice environment when establishing specific strategies to create a more productive and constructive innovation environment. Moreover, nursing managers must establish measures to ensure that nurses effectively practice IBs. This could be accomplished by reacting positively to the innovative efforts of nurses, providing them with the time and resources to undertake innovative efforts, supporting their meaningful innovative ideas, and providing incentive mechanisms and rewards for meaningful innovation. The motivational strategies that convey appreciation and acknowledgment to staff nurses must be enhanced by providing educational opportunities or financial and professional advancement.
